# Quantification of HPV16 E7 Oncoproteins in Urine Specimens from Women with Cervical Intraepithelial Neoplasia

**DOI:** 10.3390/microorganisms12061205

**Published:** 2024-06-14

**Authors:** Daiki Makioka, Mikio Inada, Masayuki Awano, Ema Saito, Takuya Shinoda, Satoko Abe, Teruki Yoshimura, Martin Müller, Toshiyuki Sasagawa, Etsuro Ito

**Affiliations:** 1 Department of Biology, Waseda University, Shinjuku, Tokyo 162-8480, Japan; mkokdik-3434@ruri.waseda.jp (D.M.); mikioinada@akane.waseda.jp (M.I.); masayuki1878@fuji.waseda.jp (M.A.); emasaito@moegi.waseda.jp (E.S.); takuya17@toki.waseda.jp (T.S.); abe3105gyotoku@outlook.com (S.A.); 2 School of Pharmaceutical Sciences, Health Sciences University of Hokkaido, Tobetsu 061-0293, Hokkaido, Japan; yosimura@hoku-iryo-u.ac.jp; 3 Tumorvirus-Specific Vaccination Strategies, Deutsche Krebsforschungszentrum (DKFZ), 69120 Heidelberg, Germany; martin.mueller@dkfz-heidelberg.de; 4 Department of Obstetrics and Gynecology, Kanazawa Medical University, Uchinada 920-0293, Ishikawa, Japan; 5 Graduate Institute of Medicine, Kaohsiung Medical University, Kaohsiung 80708, Taiwan

**Keywords:** E7 oncoprotein, human papillomavirus, noninvasive diagnosis, ultrasensitive ELISA, uniplex E6/E7 PCR, urine

## Abstract

We present the validity of using an ultrasensitive enzyme-linked immunosorbent assay (ELISA) for quantifying high-risk human papillomavirus (HPV) 16 E7 oncoproteins in urine specimens as a noninvasive method of analyzing the oncogenic activity of HPV. Some reports claim that the oncogenic activity of HPV is a more relevant clinical indicator than the presence of HPV DNA for estimating malignant potential. In the present study, urine containing HPV16 and related types were selected by uniplex E6/E7 polymerase chain reaction and classified according to the pathologic diagnosis of cervical intraepithelial neoplasia (CIN) in cervical biopsy specimens. Our ultrasensitive ELISA was able to detect attomole levels of HPV16 E7 oncoproteins, and it detected HPV16-positive SiHa cells at >500 cells/mL without detecting HPV18-positive cells. Our ELISA results showed E7 oncoproteins in 80% (4/5) of urine specimens from women with HPV16-positive CIN1, 71% (5/7) of urine specimens from CIN2 patients, and 38% (3/8) of urine specimens from CIN3 patients. Some urine specimens with undetectable E7 oncoproteins were thought to be negative for live HPV 16-positive cells or in an inactivated state of infection. These results provide the basis for assessing oncogenic activity by quantifying E7 oncoproteins in patient urine.

## 1. Introduction

According to the World Health Organization (WHO), almost all cervical cancer cases (99%) are linked to infection with high-risk human papillomavirus (HPV), an extremely common virus transmitted through sexual contact [1]. Cervical cancer is the fourth most common cancer in women globally with approximately 660,000 new cases and 350,000 deaths in 2022. The highest rates of cervical cancer incidence and mortality are in low- and middle-income countries, reflecting major inequities driven by the lack of access to national HPV vaccination, cervical screening and treatment services, and social and economic determinants [2]. HPV DNA can be detected using methods such as hybrid capture and polymerase chain reaction (PCR) [3]. The WHO endorses HPV-DNA testing as the primary screening technique for cervical cancer prevention [4]. Emerging research, however, suggests that the oncogenic activity of HPV is a more critical clinical marker of developing cervical lesions and cervical cancer than the mere presence of HPV DNA [5,6]. For example, Cuschieri et al. found that detection of HPV E6/E7 transcripts is more specific for disease diagnosis at follow-up than detection of HPV DNA, indicating that positivity for HPV mRNA transcripts at baseline significantly increases the likelihood of persistent infection over DNA detection alone [7]. Similarly, Molden and associates argue that the prognostic value of DNA testing for identifying severe dysplasia is limited, whereas HPV E6/E7 mRNA detection in combination with cytology is a more reliable prognostic indicator [8,9,10]. Additional studies highlight the occurrence of HPV DNA in cervical tissues without signs of active infection, pointing to a possible latent infection under immunologic control [11]. These insights support a nuanced screening approach that integrates DNA and mRNA testing to enhance our understanding of cervical cancer progression. Incorporating protein-level detection could significantly augment the efficacy of such a comprehensive screening strategy.

The noninvasive screening using urine instead of invasive tests, such as Pap testing, for infectious HPV will likely increase the number of people screened for HPV. Women report being more comfortable with providing urine specimens [12,13], and more attention is being focused on screening urine from cancer patients for HPV before and after treatment [14]. Therefore, it may be useful to establish a protein-level test to accurately measure the oncogenic activity of cervical cancer based on the quantification of HPV16 E7 oncoproteins in urine collected from women with cervical intraepithelial neoplasia (CIN; pathologic diagnosis of cervical biopsy specimens); for example, using an ultrasensitive enzyme-linked immunosorbent assay (ELISA) with thionicotinamide-adenine dinucleotide (thio-NAD) cycling (TN-cyclon^TM^) [15]. In the present study, we evaluated the validity of using urine in our proposed protein-level test by comparing the ELISA index (threshold of the presence of E7 oncoproteins), HPV typing with uniplex PCR (presence of E7 DNA), and the CIN grade.

We focused on HPV16 E7 oncoprotein because the pathogenesis of cervical cancer and oropharyngeal cancer is causatively associated with HPV infections, especially with HPV16 (50% of cases), HPV16-related types (types 31, 33, 35, 52, 58, and 67), and HPV18 and its related types (types 18, 39, 45, 59, 68, 70, 85, and 97) [1,16]. HPV16 and HPV18-related types are considered to be the highest-risk types, and they are thought to be the most malignant types as the causative agents of the development of cervical cancer. Therefore, we have also focused on the related types, not only HPV16 and HPV18. The E7 oncoprotein in these viruses prevents phosphorylation of the retinoblastoma protein (pRB), leading to uncontrolled cell proliferation [17], and is involved in cell transformation, mitosis, and cervical cancer cell immortalization [18,19,20]. The presence of E7 is sufficient to immortalize epithelial cells [21], and E7 has a major role in cervical cancer development in transgenic mouse models [22]. The importance of the E7 oncoprotein in the development of HPV-related malignancy demands its careful quantification in human specimens [23].

## 2. Materials and Methods

### 2.1. Specimens and Ethics

The present study was performed in Japan, and 45 urine specimens were collected from randomly selected women (24–57 years old) among women having HPV-positive CIN lesions who visited the Department of Obstetrics and Gynecology of Kanazawa Medical University Hospital, Japan, from January 2019 to December 2021. This project was approved by the Institutional Review Boards of Kanazawa Medical University (No. I450) and Waseda University (2019-325 and 2022-110). All experiments were performed in accordance with relevant guidelines and regulations, including the Declaration of Helsinki. Written informed consent was obtained from each patient at Kanazawa Medical University Hospital. A pathologist and a surgical pathologist confirmed the diagnosis for each specimen according to the WHO classification [24]. The uniplex E6/E7 PCR results and CIN information for the patients are provided in the Supplementary Materials (Table S1: Uniplex PCR results for urine specimens and CIN grades for cervical biopsy specimens for patients). When the specimens were sent to Waseda University, all information other than age, medical history, cytology, and uniplex E6/E7 PCR results was withheld to protect personal information.

### 2.2. Ultrasensitive ELISA with Thio-NAD Cycling

Specimens were processed for measurements using the ultrasensitive ELISA with thio-NAD cycling as follows: 45 mL of urine was collected from each patient and centrifuged at 3000 rpm for 5 min. The resultant precipitates were resolved with 2 mL of ThinPrep solution (Hologic, Marlborough, MA, USA) and stored at –80 °C. Our ultrasensitive ELISA with thio-NAD cycling was based on a sandwich ELISA and developed by Watabe and Ito [15]; the details are described elsewhere [25,26]. Briefly, 2 μg/mL of primary antibody (NM2) against HPV16 E7 oncoprotein, provided by an author (M.M.), in 50 mM Na_2_CO_3_ (pH 9.6) was added to each well of microplates placed at room temperature for 1 h and then blocked by incubation in 1% Tween 20 in tris-buffered saline (TBS) at room temperature for 1 h. The antigen (E7, LS-G21591; LSBio, Shirley, MA, USA) and 100 µL of the specimen were added to each well, and the mixture was incubated at room temperature for 1 h. The antigen samples were diluted with TBS containing 0.1% bovine serum albumin (BSA) to achieve a gradient concentration. BSA (0.1% in TBS) served as a blank. A 100 μL solution of the secondary antibody (NM3), which was provided for HPV16 E7 oncoprotein by an author (M.M.), conjugated with alkaline phosphatase and adjusted to 6 ng/mL in TBS containing 0.05% Tween 20 and 0.1% BSA, was added to each well of the microplates and allowed to sit at room temperature for 1 h. The antibodies, NM2 and NM3, were produced against a GST-HPV16-E7 fusion protein and used previously [27]; NM2 recognizes an epitope located between amino acids 35–60 of E7 and is an isotype IgG2a.

Finally, 100 μL of the thio-NAD cycling solution containing 10 U/mL 3α-hydroxysteroid dehydrogenase (Asahi Kasei Pharma, Tokyo, Japan), 0.4 mM 17β-methoxy-5β-androstan-3α-ol 3-phosphate, which was provided by an author (T.Y.), 1.0 mM NADH, and 2.0 mM thio-NAD in 100 mM tris-HCl (pH 9.0) was added to each well of the microplates. The detectable signal (i.e., thio-NADH) was amplified in a triangular-number manner, i.e., 1, 1 + 2, (1 + 2) + 3, (1 + 2 + 3) + 4, …, during the cycling reaction within a short time, and measured with a microplate reader (Corona Electric SH-1000, Ibaraki, Japan) at 405 nm. The 405 nm signals were normalized to those at 660 nm. The experimental data were obtained by subtracting the mean value of the blank signals from each of the corresponding measured data points.

### 2.3. Spike-and-Recovery Test

All the experimental procedures were the same as for the above ultrasensitive ELISA experiments without the antigen solution. We prepared four wells (Table 1) containing the following:

(1) 50 μL TBS with 0.1% BSA or 50 μL ThinPrep solution, this was used for the blank experiment;
(2) (50 − *x*) μL TBS with 0.1% BSA and *x* μL control urine (Serotec, Sapporo, Japan), this was also used for the blank experiment;
(3) 50 μL E7 antigen at a concentration of 0.2 pg/50 µL or 50 μL SiHa cells at a concentration of 500 cells/50 µL, this was used for the spike-and-recovery experiment;
(4) (50 − *x*) μL E7 antigen at a concentration of 0.2 pg/(50 − *x*) µL or (50 − *x*) μL SiHa cells at a concentration of 500 cells/(50 − *x*) µL, and *x* μL control urine, this was also used for the spike-and-recovery experiment.

The spike-and-recovery ratio was calculated as [absorbance of (4) − absorbance of (2)]/[absorbance of (3) − absorbance of (1)].

### 2.4. Determination of the ELISA Index

To determine whether or not specimens contain E7 oncoproteins, the index (threshold value) in the ELISA assay must be determined. This ELISA index is determined by dividing the absorbance by the blank value to account for inter-assay differences rather than by just the absorbance itself. For urine specimens, the absorbance of blank data (ThinPrep solution only) was measured three times with ELISA, and these values were averaged. The absorbance of a ThinPrep solution containing 500 cells/mL of HPV18-positive HeLa cells or HPV-negative HSC-1 cells (a squamous cell carcinoma of human skin) and 1:100 diluted urine was then measured three times with ELISA, and these values were averaged. We assumed this ThinPrep solution functioned as a control urine collected from HPV16-negative, healthy participants. The averaged value for the control specimens was divided by the blank data.

### 2.5. Uniplex E6/E7 PCR

The uniplex E6/E7 PCR was previously described in detail [28]. In brief, the uniplex E6/E7 PCR assay amplified the E6 and E7 genes of 39 common HPV types using type-specific primer pairs. This assay is capable of individually detecting 15 low-risk HPV types (HPV6, 11, 40, 42, 44, 54, 55, 61, 62, 71, 74, 81, 84, 89, and 90), 11 intermediate-risk HPV types (HPV26, 30, 34, 53, 66, 67, 69, 70, 73, 82, and 85), and 13 high-risk HPV types (HPV16, 18, 31, 33, 35, 39, 45, 51, 52, 56, 58, 59, and 68). The total volume (15 mL) of each urine specimen was centrifuged, and 2 mL of ThinPrep solution was added to the sediment. The specimens were stored at –80 °C. A 100 µL aliquot of the stored ThinPrep specimens was centrifuged, and 50 µL of alkaline lysis reagent (25 µM NaOH and 20 µM EDTA) was added to the resulting pellet. The specimens were heated at 95 °C for 15 min. Neutralization reagent (50 µL, 40 mM Tris-HCL, pH 4.5) was added to the specimens, which were diluted approximately five times with distilled water. After centrifugation, the supernatant was used as the DNA-containing solution. PCR for each HPV type was performed in a T100 thermal cycler (Bio-Rad, Hercules, CA, USA) using 10 μL of PCR mixture containing 5.0 μL of the specimen as a template, 0.5 pM of each HPV type-specific primer, and 5.0 μL of AmpliTaq Gold 360 Master Mix (Applied Biosystem, Waltham, MA, USA). A 5.0 μL aliquot of each reaction solution for each HPV type was loaded onto a 2.5% agarose gel (Bio-Rad) and electrophoresed in 1× Tris-borate-EDTA buffer for 8 min. HPV type-specific bands were visualized with SYBR Green I (Takara-Bio, Kusatsu, Japan) staining under UV light.

### 2.6. Statistical Analysis

Data are expressed as mean ± SD. The limit of detection (LOD) was estimated from the mean of the blanks, the standard deviation of the blanks, and a confidence factor of 3. The limit of quantitation (LOQ) was estimated by the same method used to estimate the LOD but with a confidence factor of 10. The coefficient of variation (CV) was obtained for the E7 antigen in examinations of the intra-assay and inter-assay reproducibility. The relationship between the absorbance of the blank and that of SiHa cells was examined by a one-way ANOVA and a post-hoc Holm test. The relationship between the ELISA index and CIN grade and between the ELISA absorbance of the blank and the ELISA absorbance of the HeLa cells were examined by a *t*-test.

## 3. Results

### 3.1. LOD and LOQ for the Measurement of HPV16 E7 Oncoproteins in a Buffer

**Figure 1:**
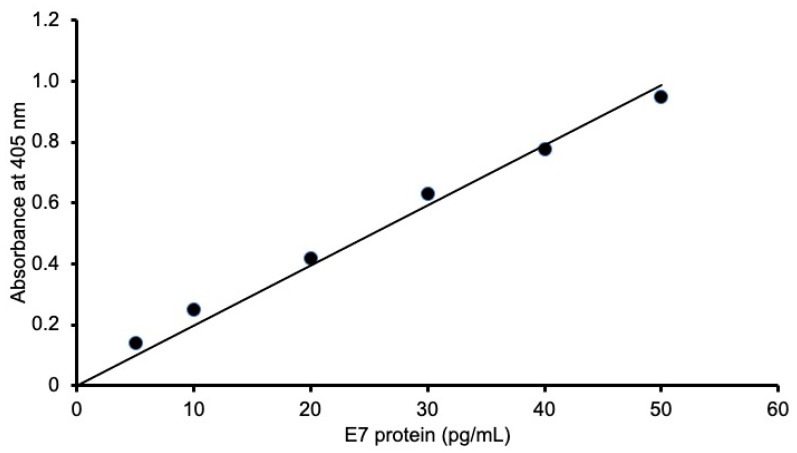
Linear calibration curve for HPV16 E7 obtained using the ultrasensitive ELISA coupled with thio-NAD cycling. This curve was calculated as the averaged values of three experiments and is expressed as *y* = 1.97 × 10^−2^*x*, *R*^2^ = 0.99 in the range of 5.00–50.0 pg/mL for the 60-min measurements. This linear calibration curve indicated an LOD of E7 of 1.18 × 10^−18^ moles/assay and an LOQ of 3.92 × 10^−18^ moles/assay. The raw data for Figure 1 are provided in Table S2 in Supplementary Materials.

To determine the LOD and LOQ for the recombinant E7 oncoprotein of HPV16 in 0.1% BSA/TBS, we produced linear calibration curves (*n* = 3) after subtracting the blank values using the data of the 60-min measurements in our ultrasensitive ELISA. The averaged curve from these three curves was expressed as *y* = 1.97 × 10^−2^*x*, *R*^2^ = 0.99 (Figure 1). The LOD of E7 was obtained as 1.18 × 10^−18^ moles/assay (i.e., 0.13 pg/mL), and the LOQ was 3.92 × 10^−18^ moles/assay (i.e., 0.43 pg/mL) when the assay volume was 100 μL and the molecular mass was 11,022 Da. These results indicate that the present method successfully detected E7 oncoproteins with ultrasensitivity [29]. The intra-assay CV was 4.2% for 16 pg/mL E7 (*n* = 3 by 1 researcher), and the inter-assay CV was 3.9% for 16 pg/mL E7 (*n* = 3 by different researchers).

### 3.2. Minimum Detection of SiHa Cells in ThinPrep Solution

**Figure 2:**
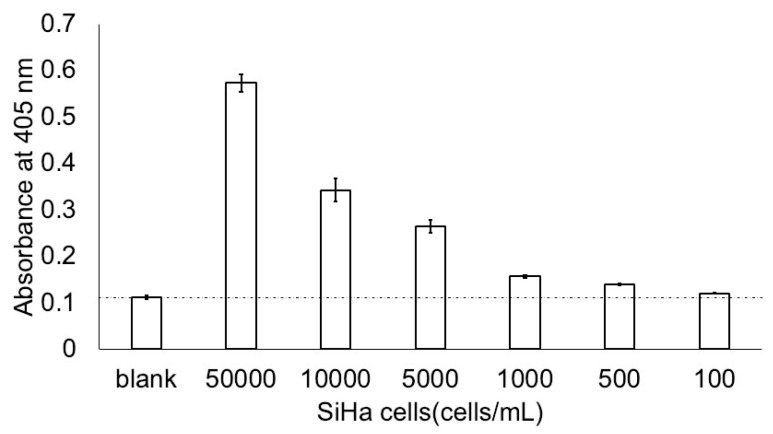
Minimum detection of SiHa cells using the ultrasensitive ELISA coupled with thio-NAD cycling. The absorbance of 500 cells/mL was larger (*p* < 0.001) than the absorbance of the blank. *n* = 3 each.

We examined the detection limit of SiHa cells sonicated in a ThinPrep solution. The measurements were performed in our ultrasensitive ELISA three times on 3 days using a diluted series of SiHa (Figure 2). The absorbance of 500 cells/mL was larger than the absorbance of the blank (500 cells/mL vs. Blank, *p* < 0.001). The intra-assay CV was 4.2% for 500 cells/mL (*n* = 3), and the inter-assay CV was 1.0% for 500 cells/mL (*n* = 3).

### 3.3. Effects of Urine on Ultrasensitive ELISA Measurements of E7 Oncoproteins and SiHa Cells

We then examined how the ultrasensitive measurement was affected by diluting the E7 oncoproteins and SiHa cells in 0.1% BSA/TBA and ThinPrep solution in control urine, respectively. The dilutions of urine were 1:50, 1:100, and 1:200. The results are shown in Table 2. The spike-and-recovery ratios for 5000 cells/mL are shown in Table 3. Accordingly, we determined that a 1:100 dilution of urine was appropriate based on the specimen amounts in the following experiments.

### 3.4. Determination of ELISA Index to Indicate Cutoff Value for the Presence of E7 Oncoproteins

The ELISA indices (threshold values) for indicating the presence of E7 oncoproteins were determined as described in the Materials and Methods section to be 1.35 and 1.30 in control urine containing HPV18-positive HeLa cells or squamous cell carcinoma HSC-1 cells, respectively.

### 3.5. Detection of E7 Oncoproteins in Urine Specimens Collected from Women with HPV16 DNA-Positive CIN Lesions

In a preliminary experiment, not only HPV16-positive but also HPV16-related types (alpha-9 group) such as HPV31, 33, 52, and 58 showed positive for anti-E7 oncoprotein antibodies used in the present assay. Out of 45 urine specimens from HPV-positive CIN women, 20 showed positive with DNA of HPV16 and its related types. A comparison of the uniplex E6/E7 PCR results for HPV16 and its related types and the CIN grade for urine specimens is shown in Table 4. As described above, when setting the ELISA index indicating the presence of E7 oncoproteins to 1.35 for HeLa cells, 4 of 5 (80%) HPV16-positive and CIN1 urine specimens contained E7, 5 of 7 (71%) CIN2 specimens contained E7, and 3 of 8 (38%) CIN3 specimens contained E7. The same results were obtained when setting the ELISA index to 1.30 for HSC1-1 cells. On the other hand, when setting the ELISA index indicating the presence of E7 oncoproteins to 1.35 for HeLa cells, 2 of 7 (29%) HPV16-negative and CIN1 urine specimens contained E7, 6 of 8 (75%) CIN2 specimens contained E7, and 9 of 10 (90%) CIN3 specimens contained E7.

### 3.6. Effects of Blood and Skin Cells on Measurements of E7 Oncoprotein by Our Ultrasensitive ELISA

We examined the effects of blood and skin cells in the urine specimens on the measurements of E7 oncoproteins by our ultrasensitive ELISA using spike-and-recovery tests with human control serum and HPV16-positive SiHa cells. The control serum was diluted with a ThinPrep solution at 1:500,000, 1:100,000, 1:50,000, 1:10,000, and 1:1000. The final concentration of SiHa cells was always 10,000 cells/mL. The spike-and-recovery ratios are shown in Table 5. The higher the blood concentration, the lower the recovery ratio. A limitation of our ultrasensitive ELISA is that blood contamination adversely affects detection.

The effects of skin cell contamination of the urine on the ultrasensitive ELISA results were also examined. We added 1000 cells/mL, 5000 cells/mL, or 10,000 cells/mL of HSC-1 cells (a cell line of a squamous cell carcinoma of human skin) to a ThinPrep solution containing 5000 cells/mL SiHa cells. The spike-and-recovery ratios are shown in Table 6. Contamination by skin cells adversely affected the detection using our ultrasensitive ELISA. Together, these results showed that urine specimens are more suitable than cervical biopsy specimens for ultrasensitive detection of E7 oncoproteins.

### 3.7. Relationship between the ELISA Index and CIN Grade

**Figure 3:**
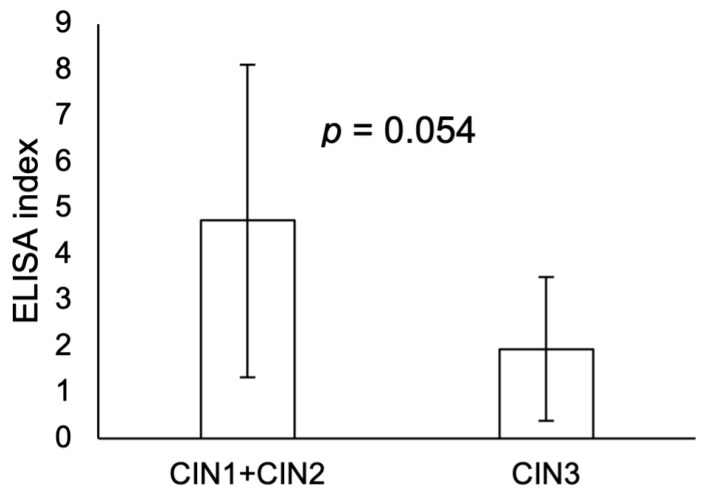
Relationship between the ELISA index and CIN grade for urine specimens. *n* = 12 for CIN1 + CIN2, and *n* = 8 for CIN3. The ELISA index showed no significant difference between CIN grades.

The ELISA index for urine specimens of women positive for HPV16 and its related types was not significant between women with a higher CIN grade and those with a lower CIN grade (Figure 3, *p* = 0.054). CIN 1 and CIN 2 were grouped because CIN1 and CIN2 have a high possibility of spontaneous recovery.

### 3.8. Specific Detection of E7 Oncoproteins in HPV16 and Its Related Types but Not HPV18-Positive Cells

**Figure 4:**
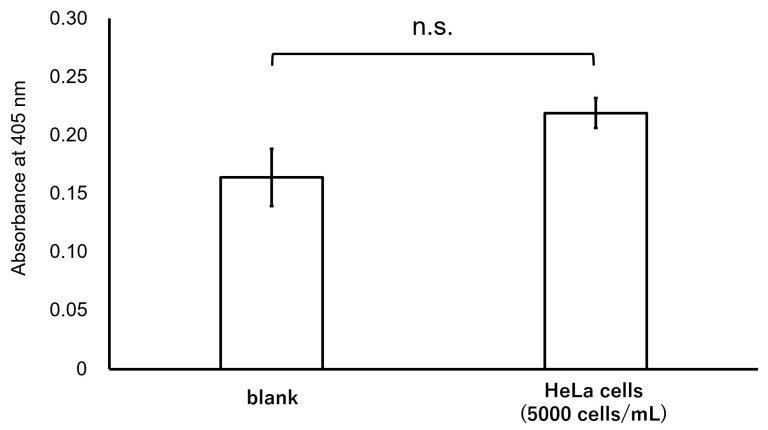
Effects of HPV18-positive HeLa cells on our ultrasensitive ELISA. No significant difference (n.s.) was detected between the absorbance of the blank and that of HeLa cells (*n* = 3 each, *p* > 0.05 by a *t*-test). That is, our ultrasensitive ELISA specifically detects HPV16 and its related type but not HPV18.

We examined whether our diagnostic system distinguishes E7 oncoproteins in HPV16 and its related types from those in HPV18. When we added 5000 cells/mL of HPV18-positive HeLa cells, a cervical carcinoma cell line containing multiple copies of integrated HPV18 DNA, to a ThinPrep solution, the detection signal of HeLa cells was almost the same as that of the blank (Figure 4). Thus, our system specifically detected HPV16 and its related types.

## 4. Discussion

The ability to precisely quantify the amount of E7 oncoproteins in specimens is critical for gaining insight into the development and progression of cervical cancer. The measurement results using our ultrasensitive ELISA system provide accurate information regarding oncogenic activity in cervical cancer, indicating the potential suitability of this system as the primary screening method, as well as mRNA testing, for a noninvasive diagnosis using urine samples. The present study demonstrated how E7 oncoproteins in high-risk HPV16 and its related types can be quantified using an ultrasensitive ELISA with thio-NAD cycling. Accurate measurements were obtained to determine the concentration of even trace amounts of E7 oncoproteins present in urine specimens collected from patients. The ultrasensitive ELISA detected attomolar levels of E7 oncoprotein and approximately 500 cells/mL of HPV16-positive SiHa cells in solution. The ELISA index, which was determined by dividing the absorbance of the sample by the absorbance of the blank, was set to determine the threshold between E7-containing and E7-non-containing specimens, allowing us to examine the relationships among the ELISA index, uniplex E6/E7 PCR DNA types, and CIN grade.

The most important finding in the present study was the discrepancy between the results of the ultrasensitive ELISA (i.e., the presence of protein) and the results of uniplex E6/E7 PCR (i.e., the presence of DNA). We consider that such a discrepancy could be explained by a variation in the HPV life cycle or in the oncogenic activity. We previously developed an ultrasensitive ELISA antigen test for use in dengue fever [26], and these studies have produced excellent results comparable to those of PCR-based tests, demonstrating that our ultrasensitive ELISA can be used to detect precise amounts of trace proteins. Therefore, it is reasonable that some specimens in the present study containing no or very low levels of E7 oncoproteins, contrary to the uniplex E6/E7 PCR-based HPV typing, represent inactive infections.

The present results demonstrated a higher prevalence of E7-positive cases with the lower-grade CIN lesions. Many studies to date have only measured mRNA, not protein, and thus the relationship between the transcription of the HPV 16 E7 oncogenes and the translation level of E7 protein levels in HPV infections is not fully established. Therefore, the present data are, to our knowledge, the first accurate research results at the protein level. As shown in Figure 3, although it is unclear due to the small number of specimens, there was a tendency for CIN1 and 2 to have a higher ELISA index than CIN3. Because many HPV researchers think that the onset of cervical carcinogenesis is intricately linked to HPV infection, specifically through the transcription of high-risk HPV E6 and E7 oncogenes, leading to elevated levels of their mRNA, the present results may be considered controversial [30,31,32]. Although the expression of E7 protein may have increased according to an increase in the CIN grade before starting the experiments, we could not confirm this because of the limited number of cases analyzed. We suggest the following two possibilities. (1) Protein expression levels may vary according to different stages of the HPV life cycle. Therefore, differences between the expression levels of E7 mRNA and E7 protein must be cautiously interpreted. A recent study demonstrated that E7 protein could not be detected while E7mRNA was highly expressed in oropharyngeal cancer, suggesting that the levels of protein expression may not reflect the levels of mRNA expression [33]. (2) Another review suggests that E6 is translated from full-length E6-E7 mRNA, whereas E7 is translated from spliced forms of E6/E7 mRNA (E6*I, E6*II, etc.) [34]. Different spliced mRNA products are likely to interfere with each other. In the viral replication stage of HPV, E7-mediated cell cycle re-entry in the middle layers of the squamous epithelium, caused by inhibition of retinoblastoma and its associated protein function, is most important for viral replication [35]. Thus, E7 is likely to have a greater influence in this step than E6.

Furthermore, the E7 oncoprotein binds to the pRB via the *N*-terminal LXCXE motif, inactivating pRB. The E7 oncoprotein of high-risk HPV promotes pRB degradation [35]. When the expression of E6 and E7 mRNAs in the basal cells increases, the cells take over many steps necessary for cancer, such as suppression of immune response, immortality, transformation, apoptosis, and suppression of differentiation. This E6/E7 mRNA increase is expected to be required for CIN2. As the progression from CIN2 to CIN3, cells derived from basal cells that highly express E6 and E7 mRNAs are also located in differentiated layers (parabasal cell layers and spiny cell layers). We believe that in CIN3 the E6 oncoproteins, rather than E7, may have an important role. Highly expressed E6 oncoproteins inactivate p53 and simultaneously suppress the expression of the *Notch1* tumor suppressor gene, and cells are thought to acquire resistance to differentiation and abnormal proliferation ability. In this regard, we believe that it is necessary to distinguish carefully between CIN2 and CIN3, and this issue will be settled by examining the quantitative change in E6 oncoprotein in the future.

Many researchers believe that the deregulation of both E6 and E7 mRNA expression plays a critical role in cancer progression after HPV DNA is integrated into the host cell genome. The expression levels of E6 and E7 may differ at different stages of the HPV life cycle. Some studies suggest that high-risk HPV E6 may more actively induce malignant progression than low-risk HPV E6, as high-risk HPV E6 inhibits p53 (inhibition of apoptosis and cell cycle deregulation of cells with DNA damage), inhibition of PDZ-binding domain proteins (disruption of cell polarity and dedifferentiation), and activation of telomerase (immortalization) [35]. Thus, E7 expression is also required, but perhaps not as much in the premalignant stage. In fact, cell proliferation is limited within the epithelium of high-grade intraepithelial lesions (CIN2, 3), whereas cell proliferation is more evident in benign HPV diseases such as condyloma. The cytotoxic T lymphocyte response to clear HPV16 infection is only attributed to its response against E6 and not E7 peptides in women with persistent HPV16 infection, as suggested by Nakagawa et al. [36]. Our team previously published data showing that anti-E6 antibodies are more prevalent than anti-E7 antibodies in CIN3 patients, even though healthy individuals do not have these antibodies, and both antibodies are detected equally in cervical cancer patients [37]. Thus, HPV16 E7 may be important in the stage of HPV replication (CIN1) but not as important in the stage of establishing high-grade lesions (CIN2, 3). Another simple explanation is that the present reaction to the E7 protein observed in CIN1 may be a reaction not only to the HPV16 E7 protein but also to E7 of other HPV16-related types, because multiple HPV-type infection was more frequently observed in CIN1 cases than in CIN3 or cancer cases. We cannot deny the possibility that, regarding the low positive rate for CIN3, it may be possible that cells are less likely to be mixed in the urine of CIN3 patients. In any event, further studies are needed to clarify this point. In the near future, we will develop our assay method to detect E6 oncoprotein expression to understand cancer progression better.

Holzinger et al., found DNA-positive and mRNA-negative samples, suggesting that latent infection samples may be present in oropharyngeal squamous cell carcinoma [38]. On the other hand, Maglennon et al., attempted to detect both DNA and proteins of rabbit oral papillomavirus in epithelial basal layers, and although they detected low levels of RNA transcripts in latently infected tissues, they did not detect late viral proteins by immunofluorescence [39]. Thus, they concluded that in a latent virus infection, the life cycle does not proceed, and the production of new viral proteins does not occur. Agreement may not necessarily exist between DNA, mRNA, and protein results. Furthermore, they demonstrated that in tissues of rabbit oral papillomavirus under latent infection, E7 and E6 mRNA levels were lower than the E2 level, which was low even during latent infection [40]. These results are not inconsistent with the fact that E5, E6, and E7 proteins are required to produce a cellular environment that supports viral DNA replication [41].

Current HPV testing is described in detail in the recent paper by Poljak et al. [42], which lists the main groups and subgroups of commercial HPV tests available on the global market in 2023: (1) high-risk HPV DNA screening test without genotyping; (2) high-risk HPV DNA screening test with concurrent partial (HPV16/18/45), concurrent extended, or reflex partial genotyping for main high-risk HPV genotypes, and we expect that the number of different tests in this category will increase remarkably, reflecting updated guidelines and changes in screening and triage practices; (3) HPV DNA full genotyping test; (4) HPV DNA genotype- or group-specific genotyping tests; (5) high-risk HPV E6/E7 mRNA tests. There may be more tests using mRNA in the future; (6) in situ hybridization HPV tests; and (7) HPV DNA tests targeting multiple non-Alpha HPV genotypes.

The possibility for self-collection of urine specimens for HPV screening is strongly desired [43,44]. A recent survey reported in a Minnesota study [45] showed an unexpectedly low rate of routine HPV screening in 21- to 29-year-old women. Reasons why some women avoid screens for cervical cancer include the following: embarrassment, fear of detrimental test results, time and cost, discomfort with male doctors, and an assumption of sexual surveillance, i.e., that cervical screenings are being used as a proxy to monitor their sexual activity [46]. As a potential solution to increase the screening rate, self-collection of cervicovaginal specimens at home with a return by mail for HPV testing is an approach that may alleviate barriers to clinic-based cervical cancer screening [47]. Although no statistically significant preference was demonstrated with urine self-sampling versus clinician sampling [48], an easy-to-use self-collection of urine may be more accommodating for examinees. Furthermore, it is necessary to consider why E7 oncoprotein is mixed in the urine. It is difficult to imagine that it enters the bloodstream from the body, passes through the kidneys, and then comes out in the urine. It is reasonable to assume that E7 oncoprotein is contained in cells that are shed from the vaginal opening along with the vaginal discharge, and that it is mixed with the urine that comes out of the urethra.

It was not possible to provide a control population of women not infected with HPV because female patients with some symptoms of cervical cancer visited the obstetrics and gynecology department of the hospital where cervical biopsy specimens were obtained and classified according to the pathologic diagnosis of CIN. The biopsy results were CIN1, 2, and 3, and then the urine specimens were collected. This means that the hospital cannot collect biopsy specimens and urine specimens from a control population of women not infected with HPV. This raises the question of whether collecting a urine specimen from a normal, healthy woman meets the control conditions, and the answer is no because there is no guarantee that the urine specimen is from a woman who is not infected with HPV as she may be asymptomatic.

As a limitation of the present study, we acknowledge that the reliability of the marker of E7 oncoprotein in urine in the diagnosis of CIN is limited due to the lack of comparison between cervical and urine specimens. Furthermore, a greater number of patient samples is needed to evaluate the presence of E7 oncoproteins from high-risk HPV strains such as HPV18. Accurate detection, however, will depend on the availability of high-quality antibodies for use in our ultrasensitive ELISA method. Due to this limitation, we cannot test for E7 oncoprotein from HPV18 in urine at this time, although we may be able to produce quality antibodies against HPV18 E7 in the future.

## 5. Conclusions

The present study establishes a foundation for the precise quantification of E7 oncoprotein in patient specimens, facilitating an in-depth examination of how E7 oncoprotein levels affect the evolution of cervical cancer precursors. Our findings highlight the potential to evaluate oncogenic activity at the protein level within the context of cervical intraepithelial neoplasia.

## Figures and Tables

**Table 1:** Protocol for the spike-and-recovery test.

	Blank		Specimen	
Solution	(1)	(2)	(3)	(4)
TBS including 0.1% BSA or ThinPrep (μL/well)	50	50 − *x*	50	50 − *x*
control urine (μL/well)	0	*X*	0	*X*
E7 antigen (0.2 pg/mL) or SiHa cells (500 cells/well)	–	–	+	+

Here, the value of *x* depends on the dilution factor of the urine. In the present experiments, three dilution factors of urine (50, 100, and 200) were considered: *x* = 2 µL for 50*x*, *x* = 1 µL for 100*x*, and *x* = 0.5 µL for 200*x*.

**Table 2:** Spike-and-recovery test using E7 antigen in urine.

Dilution Rate of Urine (%)	Spike-and-Recovery Rate (%)
50	98
100	91
200	94

**Table 3:** Spike-and-recovery test using SiHa cells (5000 cells/mL) in urine.

Dilution Rate of Urine (%)	Spike-and-Recovery Rate (%)
50	89
100	91
200	108

**Table 4:** Comparison of uniplex E6/E7 PCR results for HPV16 with CIN grade in urine specimens.

		CIN1	CIN2	CIN3	Sum
Uniplex E6/E7 PCR	Positive	5 (42%)	7 (47%)	8 (44%)	20 (44%)
	Negative	7 (58%)	8 (53%)	10 (56%)	25 (56%)
	Sum	12	15	18	45

**Table 5:** Spike-and-recovery test using SiHa cells (10,000 cells/mL) in control serum.

Dilution Time of Control Serum	Spike-and-Recovery Rate (%)
500,000	73
100,000	61
50,000	56
10,000	28
1000	9

**Table 6:** Spike-and-recovery test using SiHa cells (5000 cells/mL) with the addition of HSC-1 cells.

HSC-1 (Cells/mL)	Spike-and-Recovery Rate (%)
1000	80
5000	55
10,000	35

## Data Availability

The original contributions presented in the study are included in the article/Supplementary Materials, further inquiries can be directed to the corresponding authors.

## Supplementary Materials

The following supporting information can be downloaded at https://www.mdpi.com/article/10.3390/microorganisms12061205/s1, Table S1: Uniplex PCR results for urine specimens and CIN grades for cervical biopsy specimens for patients; Table S2: Raw data for Figure 1.
