# Supplementary material for: Quantification of HPV16 E7 Oncoproteins in Urine Specimens from Women with Cervical Intraepithelial Neoplasia
Source: Microorganisms. 2024 Jun 14;12(6):1205. doi: 10.3390/microorganisms12061205 (PMC11205804; doi:10.3390/microorganisms12061205)
Supplement: Supplementary file 1 [file microorganisms-12-01205-s001.zip › microorganisms-3018249-supplementary.pdf]

Supplementary Materials

Table S1. Uniplex PCR results for urine specimens and CIN grades for cervical biopsy specimens for patients.

Specimen number	Age	Uniplex HPV-DNA typing of urine specimens	Pathologic diagnosis
U1	53	18,51	CIN2
U2	24	53	CIN1/VAIN
U3	32	16	CIN2/3
U4	30	-	CIN3
U5	28	-	CIN2/3
U6	47	-	CIN1/3
U7	35	-	CIN1
U8	29	-	CIN2/3
U9	30	-	CIN1/2
U10	27	16,58	CIN2/3
U11	53	66	CIN2
U12	24	-	CIN2
U13	32	58	CIN1/2
U14	30	-	CIN2
U15	28	58	CIN1/2
U16	47	-	CIN1
U17	35	18,58	CIN2/3
U18	45	58	CIN2/3
U19	35	56	CIN1 susp.
U20	36	-	CIN2/3
U21	49	-	CIN2
U22	40	33.67	CIN2/3
U23	49	-	CIN3
U24	37	-	CIN3
U25	34	52	CIN1
U26	36	-	CIN2/3

U27	44	16,56,58,66	CIN1
U28	31	53,66	VAIN1 susp.
U29	23	53	CIN2/3
U30	39	16,56	CIN1
U31	33	52	CIN 2/3
U32	20	52,53,56	CIN1/2
U33	49	39,59	CIN2
U34	34	16,18	VAIN2
U35	28	31,52,53	CIN2/3
U36	51	53,56	CIN 1/2
U37	57	53,56	CIN1
U38	39	16	CIN 2/3
U39	48	53,56,58,66	CIN 1/2
U40	33	31,56,66	CIN 2
U41	46	35	CIN 1/2
U42	24	31,52	CIN 1
U43	45	31,53	CIN 1
U44	34	56	Condyloma
U45	33	-	CIN2/3

Minus indicates that the unilpex PCR results are negative.

Table S2. Raw data for Figure 1.

Absorbance of blank					
0.031	0.026	0.026	0.034	0.030	0.029
Absorbance of 5 pg/mL					
0.153	0.134	0.136			
Absorbamce of 10 pg/mL					
0.257	0.239	0.231			
Absorbance of 20 pg/mL					
0.452	0.407	0.400			
Absorbance of 30 pg/mL					
0.679	0.623	0.594			
Absorbance of 40 pg/mL					
0.808	0.770	0.752			
Absorbance of 50 pg/mL					
1.005	0.939	0.900			
